# Frequency of emergency department visits and hospitalizations due to chronic obstructive pulmonary disease exacerbations in patients included in two models of care

**DOI:** 10.7705/biomedica.4815

**Published:** 2019-12-30

**Authors:** Abraham Alí, Luis Fernando Giraldo-Cadavid, Elizabeth Karpf, Luz Adriana Quintero, Carlos Eduardo Aguirre, Emily Rincón, Alma Irina Vejarano, Ivonne Perlaza, Carlos A. Torres-Duque, Alejandro Casas

**Affiliations:** 1 Departamento de Investigación, Fundación Neumológica Colombiana, Bogotá, D.C., Colombia Departamento de Investigación Fundación Neumológica Colombiana BogotáD.C Colombia; 2 Facultad de Medicina, Universidad de La Sabana, Bogotá, D.C., Colombia Facultad de Medicina Universidad de La Sabana BogotáD.C Colombia

**Keywords:** Pulmonary disease, chronic obstructive, symptom flare up, emergencies, hospitalization, program evaluation, cohort studies, enfermedad pulmonar obstructiva crónica, brote de los síntomas, urgencias médicas, hospitalización, evaluación de programas y proyectos de salud, estudios de cohortes

## Abstract

**Introduction:**

Exacerbations of chronic obstructive pulmonary disease (COPD) have a huge impact on lung function, quality of life and mortality of patients. Emergency Department visits and hospitalizations due to exacerbations cause a significant economic burden on the health system.

**Objective:**

To describe the differences in the number of emergency visits and hospitalizations due to exacerbations of COPD among patients included in two models of care of the same institution.

**Materials and methods:**

A historical cohort study in which COPD patients who are users of two models of care were included: COPD integrated care program (CICP) and general consultation of pulmonology (GCP). The first model, unlike the second one, offers additional educational activities, 24/7 telephone service, and priority consultations. The number of emergency visits and hospitalizations due to COPD exacerbations in patients who had completed at least one year of follow-up was evaluated. The multivariable Poisson regression model was used for calculating the incidence rate (IR) and the incidence rate ratio (IRR) with an adjustment for confounding factors.

**Results:**

We included 316 COPD patients (166 from the CICP and 150 from the GCP). During the year of follow-up, the CICP patients had 50% fewer emergency visits and hospitalizations than patients from the GCP (IRR=0.50, 95%CI: 0.29-0.87, p=0.014).

**Conclusions:**

COPD patients in the CICP had fewer emergency visits and hospitalizations due to exacerbations. Prospective clinical studies are required to confirm the results and to evaluate the factors that contribute to the differences.

## Resumen

**Introducción.:**

Las exacerbaciones de la enfermedad pulmonar obstructiva crónica (EPOC) tienen un gran impacto en la función pulmonar, la calidad de vida y la mortalidad de los pacientes. Las visitas al Departamento de Emergencias y las hospitalizaciones debido a las exacerbaciones, causan una carga económica importante para el sistema de salud.

**Objetivo.:**

Describir las diferencias en el número de visitas de emergencia y hospitalizaciones debidas a exacerbaciones de la EPOC, entre los pacientes incluidos en dos modelos de atención de la misma institución.

**Materiales y métodos.:**

Se trata de un estudio de cohorte histórica en el que se incluyeron pacientes que son usuarios de dos modelos de atención: el programa de atención integrada de la EPOC (CICP) y la consulta general de neumología (PCG). El primer modelo, a diferencia del segundo, ofrece actividades educativas adicionales, servicio telefónico las 24 horas del día y consultas prioritarias. Se evaluó el número de visitas de emergencia y hospitalizaciones debido a exacerbaciones de la EPOC en pacientes que habían completado, al menos, un año de seguimiento. Se utilizó el modelo de regresión multivariable de Poisson para calcular la tasa de incidencia (IR) y la razón de tasas de incidencia (IRR), con un ajuste para factores de confusión.

**Resultados.:**

Se incluyeron 316 pacientes con EPOC, 166 del CICP y 150 de la PCG. Durante el año de seguimiento, los pacientes en el CICP tuvieron 50 % menos visitas de emergencia y hospitalizaciones que los pacientes en la PCG (IRR=0,50; IC95% 0,29-0,87; p=0,014).

**Conclusiones.:**

Los pacientes con EPOC en el CICP, tuvieron menos visitas de emergencia y hospitalizaciones debido a las exacerbaciones. Se requieren estudios clínicos prospectivos para confirmar los resultados y evaluar los factores que contribuyen a las diferencias.

Chronic obstructive pulmonary disease (COPD) is a globally prevalent entity ^1^ with a high underdiagnosis rate ^2^; it is estimated that COPD will be the fifth world’s leading cause of death by 2030 ^3^. In people older than 40 years, COPD has a prevalence of 14.5% in Latin America ^4^ and 8.9% in Colombia ^5^. It is generally caused by cigarette smoke ^6^^,^^7^, but in emerging countries, particularly in Colombia, exposure to wood smoke has a crucial etiological role ^5^^,^^8^. According to estimations of the *Departamento Administrativo Nacional de Estadística* (DANE), of the total deaths in the country in 2010, 4,500 (2.25%) were due to respiratory diseases affecting the lower airways, secondary to tobacco consumption ^9^.

COPD is frequently progressive and is not confined to the lungs, it also produces systemic manifestations ^7^^,^^10^. These characteristics frequently make COPD a disabling disease with a high individual, family, social and economic impact and have led, in the last two decades, to the creation of integrated care programs as a measure to improve the comprehensive care of patients ^11^. Integrated care and self-management programs have contributed to improve the quality of life and exercise capacity; and to reduce hospitalizations in COPD patients with moderate and severe obstruction ^12^. In the context of primary care, although some studies have shown an improvement in the quality of life ^13^, the impact of these programs has been less clear ^14^. In Europe, the management of COPD patients in an integrated care program showed a reduction in hospitalizations due to exacerbations in comparison with usual care ^15^^-^^17^.

Exacerbations of COPD requiring hospital admission occur across all stages of airflow limitation and are a significant prognostic factor of reduced survival across all COPD stages. Patients with COPD at a high risk for hospitalization can be identified by their past history for similar events ^18^.

The present study was designed to evaluate the differences in the number of emergency visits and hospitalizations due to exacerbations in COPD patients from the same institution included in two models of care, one of them being an integrated care program.

## Materials and methods

### Design and collection of information

We designed an analytical historical cohort study. We included COPD patients who were users of one of two models of care and were followed for at least one year: COPD integrated care program (CICP) and general consultation of pneumology (GCP).

The two models belong to the same institution specialized in respiratory medicine (*Fundación Neumológica Colombiana*, FNC), located in Bogotá, which mainly serves patients of the contributory regimen in the national social security system. The GCP started on 1992 and the CICP started on 2005. To allow comparability, only patients from the *Plan Obligatorio de Salud* (name in force during the time of data collection) who had completed at least one year of follow-up between 2011 and 2015 were included.

The inclusion of patients was made for convenience, based on the completeness of the information (1 year of follow-up), until fulfilling the calculated sample. We reviewed the clinical records and institutional databases. The completeness of information referred only to a complete year of follow-up (patients having some missing information but having a complete year of follow-up were not excluded). The convenience sampling of patients, including the requirement about the completeness of the information, worked equally for both groups (CICP and CGP) to decrease the risk of a disbalance between groups in this regard.

Patients were assigned to the CICP or the CGP depending on the contracts of the insurers that pay for their care (some insurers pay for the CICP and others just pay for the GCP), such assignment remained unchanged during the study period for all recruited subjects. To compensate the risk of selection and confusion biases related with this method of assignment we used multivariable analysis (see further details in statistical analysis).

Patients older than 40 years, having at least one year of follow-up, were enrolled and the diagnosis of COPD was confirmed by the verification of the postbronchodilator forced expiratory volume in one second (FEV1) over the forced vital capacity (FVC) ratio (FEV1/FVC) lower than 0.7 and a consistent history of exposure to a risk factor (smoking, wood smoke or occupation).

The study was presented and approved by the ethics committee of the *Fundación Neumológica Colombiana* and was considered a research without risk according to Colombian regulations.

### Models of care

Both the CICP and the GCP offer education, prevention, comprehensive treatment, and rehabilitation. Unlike the GCP, the CICP is interdisciplinary (pulmonologist, internist, respiratory therapist, psychologist and nutritionist), and offers additional educational activities (individualized reinforcement training in the use of inhalers, the importance of medication compliance, suggested nutrition and physical activity among others), 24/7 telephone service and priority consultations (made by pulmonologists that are performed on the same day the patient calls describing deterioration of their respiratory condition).

The characteristics of the medical consultation and the group of medical specialists who care the patients are similar. Patients receive an average of six consultations per pulmonologist in one year, six evaluations and educational activities per respiratory therapist, two consultations for nutrition, two consultations for psychology, medical consultations in need and permanent telephone support in case of doubts or decompensation.

The prescription of physical activity and pulmonary rehabilitation, as well as the guidelines that guided pharmacological management were also similar in both groups [ALAT COPD Guideline (2011) ^19^, Colombian COPD Guideline (2013) and GOLD Strategy (2011-2015) ^21^]. Patients were assigned to the CICP or the CGP depending on the contracts of the insurers that pay for their care, and this has a risk of selection bias that we seek to reduce through multivariable analysis. The patients in the GCP group could access the interdisciplinary team if the pulmonology makes an interconsultation.

### Measurements

Information on demographic variables (gender, age, marital status) risk factors (tobacco use, wood smoke), respiratory symptoms, comorbidities (hypertension, diabetes, coronary heart disease, thromboembolic disease, congestive heart failure, sleep apnea, cancer), spirometry measurements (FEV1/FVC, FEV1) and six-minute walk test (6MWT) was included during the year of follow-up.

The type of pharmacological treatment was also recorded, use of shortacting bronchodilators, long-acting bronchodilators, inhaled steroids and phosphodiesterase inhibitors.

The number of emergency visits and hospitalizations due to exacerbations of COPD was evaluated in patients who had completed at least one year of follow-up.

### Definition of severity and exacerbations in COPD

The severity of COPD was graded according to the Colombian Clinical Practice Guideline on COPD ^20^, which takes into account the degree of dyspnea, the degree of obstruction and the frequency of exacerbations or hospitalizations in the previous year.

Exacerbations were defined as ‘mild’ if they were managed on an outpatient basis only with adjustment of bronchodilator treatment; ‘moderate’ if they required the use of systemic corticosteroids and/or antibiotics without the need for emergency visits or hospitalization; and ‘severe’ if they required an emergency visit for more than 24 hours or admission ^22^.

The emergency visits and hospitalizations were evaluated by self-report: in every follow-up consultation, patients were interrogated for the number of visits to the emergency room or hospitalizations, and this information was immediately recorded in the patient’s database (we did not use other institutions databases).

### Statistical análisis

A sample size of 290 patients was calculated using the Whitehead algorithm ^23^ to determine sample sizes for comparing two counts of events, to detect an incidence rate ratio (IRR) of exacerbations of 0.7 (30% reduction in risk) by comparing the intervention group (CICP) versus the GCP in a Poisson regression model, with 80% power, 95% confidence (two-tailed) and an expected exacerbation rate of 0.89 per patient per year in the GCP ^23^^,^^24^.

For continuous variables with normal distribution (according to the Kolmogorov-Smirnov test) means and standard deviations were used, otherwise (non-normal distribution) medians and interquartile ranges were used. For categorical variables proportions were calculated, and comparisons were made using the chi-square test, evaluating their statistical significance. The p value for statistical significance was the same for all statistical análisis (p<0.05, two-tailed).

For the analysis of the number of exacerbations according to their severity, the incidence rate (IR) per year [number of events/(person-years at risk)] was calculated for each model of care: CICP and GCP and an IR ratio (IRR) was measured. A Poisson multivariable regression model was used to compensate for potential selection and confusion biases related to the recruitment method (convenience sampling) and the observational nature of this study. The dependent variable of the Poisson regression model was the number of events, the exposure variable was the person-years at risk.

For the offset variable, we used the “*exposure*” option included in Stata™ with the variable representing exposure (person-years at risk), in such case Stata™ takes the log of exposure (the offset variable represents the log of exposure).

The potentially confounding variables assessed in our multivariable analyses included differences between both groups in COPD severity (COPD severity classification, severity of functional compromise and distance in sixminute walk test), medications influencing the risk of exacerbations (LABA, LAMA, corticosteroids, roflumilast, chronic oxygen therapy and pulmonary rehabilitation), demographic characteristics (body mass index, age, sex and marital status) and comorbidities (congestive heart failure, tobacco exposure, hypertension, diabetes, thromboembolic disease, sleep apnea and cancer).

The severity of COPD was established according to the Colombian Clinical Practice Guideline on COPD ^20^, use of long-acting bronchodilators, use of inhaled corticoids and number of comorbidities.

Bivariate analyses were performed looking for associations between potential confounders and the intervention group and also for associations between such potential confounders and the dependent variables. Potential confounders included sociodemographic, clinical and treatment variables that, due to biological plausibility, could be associated with the intervention or with the effect.

A p value <0.2 was used to select potential confounders to be introduced in the multivariable models, such a p value was higher than the p value of 0.05, used for statistical significance (see above), as recommended ^25^ {Hosmer DW, 2013 #428}, in order to avoid missing any confounding variable.

Those variables that in the bivariate analyses were associated with a p<0.2 with the intervention and the dependent variable under study and that were not mediator variables ^26^ were introduced into the multivariable model to build a saturated model. Afterwards, those variables not reaching a statistically significant association with the dependent variable (p>0.05) in the saturated model and that when eliminated did not significantly affect the regression coefficients or R2 were removed from the model, to leave it as parsimonious as possible.

The statistical analysis was performed using SPSS™ statistical software, version 22, Stata™, version 11, and MedCalc™, version 16.

## Results

Three hundred and sixteen COPD patients, 166 from the CICP and 150 from the GCP were included. Table 1 shows the demographic and clinical characteristics of the participants. The mean age was similar in both groups, with a male proportion of 53.6% in CICP and 64.7% in GCP. The participants in the two groups were similarly distributed for the variables of active smoking, severity of functional compromise, value in liters and percentage of post-bronchodilator FEV1 in spirometry, six-minute walk test, comorbidities and participation in pulmonary rehabilitation programs. The follow-up time was of one year for all patients. The outcomes were the numbers of events (hospitalization, emergency room (ER) visits and exacerbation not requiring ER visit or hospitalizations). Such events had a Poisson distribution and their standard deviation values were lower than the mean values. Therefore, the best way of analyzing them was using Poisson regression.

**Table 1:** Characteristic of patients

Characteristic	CICP group		GCP group		p value
Age, years		76 ±9	77±10		0.32
Males	89	(53.6)	97	(64.7)	0.46
BMI*	25.64±4.6		26.79±5.5		0.045
Exposure to tobacco	126	(84)	84	(67.7)	0.002
Exposition to wood smoke	48	(28.9)	51	(43.6)	0.011
Active smoking	16	(9.6)	10	(7.2)	0.45
Classification of COPD severity**					
Mild	4	(2.4)	2	(1.3)	0.68
Moderate	56	(36.7)	95	(63.3)	<0.001
Severe	75	(45.2)	34	(22.7)	<0.001
Very severe	31	(18.7)	19	(12.7)	0.14
Severity of functional compromise***					
Mild	11	(7.1)	13	(10.1)	0.27
Moderate	98	(63.2)	86	(66.7)	0.89
Severe	37	(23.9)	26	(20.2)	0.83
Very severe	9	(5.8)	4	(3.1)	0.97
Post-bronchodilator FEV1- % predicted,		58±16	62±20		
Post-bronchodilator FEV1/FVC	49.84 ± 13		55.65 ± 14.2		<0.001
6-minute walk test, meters	407 ± 134		404 ± 111		0.86
Comorbidities					
Arterial hypertension	89	(53.6)	85	(56.7)	0.58
Type 2-diabetes mellitus	25	(15.1)	18	(12)	0.42
Coronary heart disease	23	(13.9)	21	(14)	0.97
Thromboembolic disease	10	(6)	4	(2.7)	0.14
Congestive heart failure	9	(5.4)	20	(13.3)	0.015
Sleep apnea	50	(30.1)	37	(24.7)	0.27
Cancer	17	(10.2)	11	(7.3)	0.36
Treatment					
Long-term oxygen therapy	119 (71.7)		124	(83.2)	0.015
Pulmonary rehabilitation	47	(28.3)	35	(23.3)	0.31
Short-acting bronchodilators	82	(49.4)	76	(50.7)	0.82
Long-acting bronchodilators	157	(94.6)	122	(81.3)	<0.001
Inhaled steroids	100	(60.2)	90	(60)	0.96
Roflumilast	5	(3)	3	(2)	0.72

Values presented as means ± standard deviations or n (%)

Categorical variables were assessed using chi square or Fisher test in case of low frequencies.

Categorical or quantitative variables that were associated with p<0.2 were introduced in the saturated multivariable model.

* BMI: Body Mass Index

** Classification of severity according to the Colombian Guide to Clinical Practice in COPD(20)

*** Severity of functional compromise: categorization of FEV1 pos%. Mild >80%, moderate 50-80%, severe 30-49% and very severe <30%.

Significant differences were found in BMI, tobacco and biomass exposure, severity of COPD, post-bronchodilator FEV1/FVC ratio, concomitant congestive heart failure and treatment with chronic home oxygen treatment and long-acting bronchodilators (table 1). The post-bronchodilator FEV1/FVC ratio was 49.8% (±13) in the CICP group and 55.6% (±14.2) in the GCP group. The use of long-acting bronchodilators was more frequent in the CICP group than in the GCP, and chronic home oxygen treatment in the GCP group. The frequency of exacerbations by program is presented in the table 2, with median and interquartile range.

**Table 2:** Frequency of exacerbations by program

				Program			
							
		CICP				GCP	
	n	Median		IQR	n	Median	*IQR*
	
Number of mild exacerbations	34	0	0 - 0		50	0	0 - 1
Number of moderate exacerbations	91	0	0 - 1		26	0	0 - 0
Number of severe exacerbations	56	0	0 - 0		64	0	0 - 1
Number of mild plus moderate exacerbations	125	0	0 - 1		76	0	0 - 1
Number exacerbations (Total)	181	1	0 - 2		140	0	0 - 1

CICP: COPD integrated care program; GCP: general consultation of pulmonology; IQR: interquartile range (percentile 25- percentile 75)

Follow-up time: 1 year for all patients.

After the adjustment by confounding variables, a lower number of severe exacerbations (those requiring emergency visits for more than 24 hours or hospitalizations) were found in the CICP group versus the GCP group (IR: 21.4 vs. 42.7 per 100 person-years) with an IRR of 0.50 (95% CI: 0.29-0.87, p=0.014), which means a 50% reduction in the use of these health resources by CICP patients (Table 3). Similarly, the analysis of the emergency visits only showed a lower number in the CICP group versus the GCP group (IR: 9.7 vs. 20.7 per 100 person-years, respectively); with an IRR of 0.47 (95% CI: 0.26-0.85; P=0.012), which means a 53% reduction in emergency visits in the CICP group (table 3).

**Table 3:** Incidence rate and incidence rate ratio of severe exacerbations per model of care

	CICP	GCP	Adjusted IRR	
Dependent variable			(multivariable model)	p value
	
	IR	IR	IRR (IC 95%)	
				
Visits to emergency department	9.7	20.7	0.47 (0.26-0.85)	0.012
Hospitalizations	17.5	20.7	0.85 (0.52-1.39)	0.510
Severe exacerbations	21.4	42.7	0.50 (0.29-0.87)	0.014
Moderate exacerbations	63.3	17.3	3.65 (1.46-9.14)	0.006
Mild exacerbations	14.5	33.3	0.44 (0.27-0.70)	0.001
Mild plus moderate exacerbations	65.4	50.7	1.29 (0.92-1.73)	0.089

CICP: COPD integrated care program; IR: incidence rate (adjusted rates); IRR: incidence rate ratio (adjusted IRR)

* The 316 patients are in the model shown. Estimates for incidence rate in the multivariable analysis were obtained by a Poisson regression model. IC stands for confidence interval.

The IR for each group were the number of events (emergency services visits, hospitalization, severe, moderate or mild exacerbations) per 100 person-years.

Follow-up time: 1 year for all patients.

In contrast, a higher number of moderate exacerbations (were found in the CICP group versus the GCP group (IR: 63.3 vs. 17.3 per 100 personyears, respectively) with an IRR of 3.65 (95% CI: 1.46-9.14, p=0.006) (Table 3). There were no differences between the groups for hospitalizations due to exacerbations (table 3).

Other factors that were independently associated with an increased risk of exacerbations were a greater severity of COPD for all types of exacerbations (p<0.01). Long-acting bronchodilator use was associated with a lower risk of severe exacerbations (p<0.001) and the fact of being unmarried for severe exacerbations (p=0.019). Age, BMI, the presence of comorbidities and the risk factor for COPD (wood smoke vs. smoking) were not independently associated with the frequency of COPD exacerbations. The detailed results of the multivariable analysis with the variables included in the more parsimonious models are shown in the supplementary appendix**.**

## Discussion

During the year of follow-up, CICP patients had 50% fewer severe exacerbations (IRR=0.50, p=0.014) and 53% fewer emergency visits (IRR=0.47, p<0.012) in comparison with GCP patients, although the proportion of patients with severe COPD was higher in the CICP group. Given that the rate of mild plus moderate exacerbations was not significantly different between the CICP and GCP, and that the number of outpatient visits increased in CICP patients, it is possible that having 24/7 telephone service and priority consultations to CICP patients may have influenced the lower number of emergency visits and hospitalizations in this group; but this requires a confirmatory prospective study. Although patients of both programs were routinely asked about exacerbations in every follow-up consultation and such data were immediately recorded, GCP patients had a less close follow-up, and that is one of the points to take into account to explain the differences in results.

It has been previously documented that integrated care and selfmanagement programs improve quality of life and exercise capacity and reduce hospitalizations in COPD patients with moderate and severe obstruction ^12^. Although integrated care also includes pulmonary rehabilitation programs whose benefits in exercise capacity, dyspnea and quality of life are equally well established, especially in patients with moderate and severe COPD ^27^^,^^28^, we believe that the additional components of the program play a role in the benefits obtained. In addition to the usual care and training, emphasis on self-management of the disease is made to COPD CICP patients in this study through additional educational activities; CICP patients are also provided with having 24/7 telephone service and priority consultations that are not possible through the GCP.

In cases of exacerbation, CICP patients receive timely and intensive outpatient treatment, preventing the progression of the exacerbation, visits to emergency department and hospitalizations. This finding is related to what has been described in the United Kingdom, where rapid recognition and treatment improve recovery and reduce the risk of hospitalization ^29^. A recent study showed that a disease management program based on a multidisciplinary approach is useful in patients with impaired exercise capacity and little obstruction in spirometry ^30^. This reduction of emergency visits and hospitalizations is essential in the management of patients, not only because of its clinical impact at an individual level but also because of the significant reduction of care costs ^16^^,^^17^^,^^31^.

Although in this study the proportion of patients receiving treatment with long-acting bronchodilators was higher in the CICP group, the CICP intervention continued to be independently associated with a lower risk of severe exacerbations after adjusting for this confounding variable in the Poisson multivariable regression model. However, this finding should be highlighted because in recent years the use of long-acting bronchodilators, alone (monotherapy) or in combination (double therapy), has become the first line of treatment, and its utility has been proven in improving symptoms and reducing exacerbations^7^^,^^32^^,^^33^.

More than 80% of the patients in the two groups of our study are treated with long-acting bronchodilators. This behavior is not repeated in patients managed outside specialized institutions, which has been corroborated in Colombia, where the formulation of short-acting bronchodilators is still predominant ^34^. Although, the period of inclusion of patients for this study (2011-2015) precedes much of the information on long-acting bronchodilator treatment in COPD, the guidelines in force at that time already highlighted the role of anticholinergics and long-acting beta-agonists, indicating lack of adherence to national and international guidelines, mainly for administrative and cost reasons (lack of inclusion of drugs in the mandatory health plan).

Other factors that may be related to the finding of fewer COPD exacerbations in CICP patients are not apparent. There were no significant differences between groups regarding age, comorbidities, and participation in pulmonary rehabilitation programs (includes exercises, energy saving activities, dance, Tai Chi, pre-established educational workshops).

There is not a clear explanation for the difference in exposure to tobacco and biomass (higher proportion of exposure to biomass in GCP patients), and it does not appear to have an impact on the difference in emergency visits and hospitalizations (the results were not modified when adjusted for this variable in the Poisson multivariable regression model).

The higher proportion of patients with severe COPD included in CICP can be understood because health insurers select this group of patients to refer them to a structured program in a specialized institution. It is expected that patients in a more severe condition will have a higher frequency of visits to emergency department and hospitalizations due to COPD exacerbations. In this study, there was a higher proportion of patients in a severe condition in CICP. The fact that CICP patients had fewer emergency visits and hospitalizations, despite their more severe disease, highlights the magnitude of the difference.

The main limitation of this study is its historical cohort design. Recall bias may have affected outcomes, but it would be expected that the group with a less close follow-up (GCP) would have reported fewer exacerbations due to this bias, which would have harmed (instead of favoring) the results of the intervention. Both groups were equally treated regarding the requirement of including only patients having a complete year of follow-up, such requirement aimed at having comparable groups, we did not excluded patients with some missing information in their medical records. Therefore, we do not think that our study has an important risk of bias related to including only patients with complete medical records.

This type of studies can be affected by confounding variables, although an extensive multivariable analysis was conducted (see the statistical analysis section and the supplementary appendix) to control for confounding variables, the effect of an unmeasured confounding variable cannot be ruled out. There were discrete differences in the medications used in each of the groups, but we adjusted for this variable in the multivariable analysis, and we consider that it did not impact the results obtained. All this shows that it would be advisable to confirm its results through randomized clinical trials. As happens in any integrate care program (composed of various interventions administered simultaneously) it is not possible to determine which of the interventions of the CICP is responsible of the differences in the outcomes.

In conclusion, in this historical cohort study, patients included in CICP had about 50% fewer visits to emergency department and hospitalization for severe exacerbations during one year of follow-up compared to GCP patients in the same institution. These results justify a randomized clinical trial to confirm them.
